# Preclinical evaluation of the gorilla‐derived HAdV‐B AdV‐lumc007 oncolytic adenovirus ‘GoraVir’ for the treatment of pancreatic ductal adenocarcinoma

**DOI:** 10.1002/1878-0261.13561

**Published:** 2024-01-24

**Authors:** Selas T. F. Bots, Tom J. Harryvan, Christianne Groeneveldt, Priscilla Kinderman, Vera Kemp, Nadine van Montfoort, Rob C. Hoeben

**Affiliations:** ^1^ Department of Cell and Chemical Biology Leiden University Medical Center The Netherlands; ^2^ Department of Gastroenterology and Hepatology Leiden University Medical Center The Netherlands; ^3^ Department of Medical Oncology Leiden University Medical Center The Netherlands

**Keywords:** CD46, non‐human primate oncolytic adenovirus, pancreatic ductal adenocarcinoma, tumour‐stroma, xenograft model

## Abstract

Pancreatic ductal adenocarcinoma (PDAC) is a highly aggressive malignancy which shows unparalleled therapeutic resistance due to its genetic and cellular heterogeneity, dense stromal tissue, and immune‐suppressive tumour microenvironment. Oncolytic virotherapy has emerged as a new treatment modality which uses tumour‐specific viruses to eliminate cancerous cells. Non‐human primate adenoviruses of the human adenovirus B (HAdV‐B) species have demonstrated considerable lytic potential in human cancer cells as well as limited preexisting neutralizing immunity in humans. Previously, we have generated a new oncolytic derivative of the gorilla‐derived HAdV‐B AdV‐lumc007 named ‘GoraVir’. Here, we show that GoraVir displays oncolytic efficacy in pancreatic cancer cells and pancreatic‐cancer‐associated fibroblasts. Moreover, it retains its lytic potential in monoculture and co‐culture spheroids. In addition, we established the ubiquitously expressed complement receptor CD46 as the main entry receptor for GoraVir. Finally, a single intratumoural dose of GoraVir was shown to delay tumour growth in a BxPC‐3 xenograft model at 10 days post‐treatment. Collectively, these data demonstrate that the new gorilla‐derived oncolytic adenovirus is a potent oncolytic vector candidate that targets both pancreatic cancer cells and tumour‐adjacent stroma.

## Graphical abstract

Oncolytic adenovirus ‘GoraVir’ has strong lytic potential in both pancreatic cancer cells and cancer‐associated fibroblasts, which may be facilitated by its use of CD46 for viral entry. Moreover, GoraVir demonstrates superior oncolytic efficacy compared to HAdV‐C5 in spheroid cultures as well as *in vivo*. This shows that GoraVir exhibits unique oncolytic properties and seems a promising candidate for the treatment of pancreatic cancer.

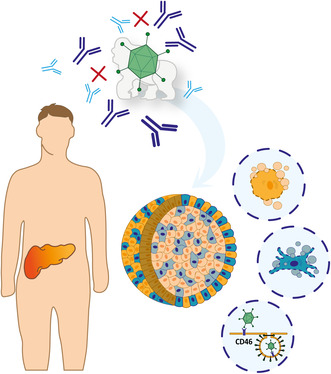

**Abbreviations**
CAF: cancer‐associated fibroblast
CAR: coxsackie and adenovirus receptor
EV: empty vector
hAd: human adenovirus
HAdV‐C5: human adenovirus type 5
KO: knock‐out
nhpAd: non‐human primate adenovirus
OVT: oncolytic virotherapy
PDAC: pancreatic ductal adenocarcinoma
TME: tumour microenvironment
WT: wildtype

## 1 Introduction

Pancreatic ductal adenocarcinoma (PDAC) is a highly aggressive malignancy with a five‐year overall survival rate of around 10% (National Cancer Institute). The majority of patients presents with unresectable, locally advanced, or metastatic disease at the time of diagnosis which prohibits curative surgery. Moreover, PDAC shows unrivalled therapeutic resistance due to its genetic and cellular heterogeneity, dense stromal tissue, and immune‐suppressive tumour microenvironment (TME) [1]. The promising results of immunotherapies in other types of malignancies led to pre‐clinical and clinical studies in PDAC (*e.g*., checkpoint inhibitors and therapeutic vaccines) both as standalone treatments as well as in combinatorial approaches [2]. Unfortunately, most of these showed only moderate improvements in survival. Hence, there is a need for improved immunotherapies that can overcome the inhibition by the immunosuppressive TME in PDAC [2].

Oncolytic virotherapy (OVT) has emerged as a new anti‐cancer treatment and comprises the use of viruses that selectively infect, replicate in, and kill cancerous cells as opposed to healthy cells. Upon virus‐induced cell death virus progeny is released, as well as danger‐ and pattern‐associated molecular patterns, and tumour (neo)antigens. Together, these pro‐inflammatory molecules can initiate an immune response directed against virus‐infected cells as well as against the tumour cells [3]. OVT might be especially powerful to overcome difficulties posed by the genetic and cellular heterogeneity in PDAC as it harnesses a diverse range of anti‐tumour activities that could be reactive to a broad range of cancer cells. Human adenoviruses (hAds) are one of the candidate viruses employed in OVT and have shown both efficacy and tolerability in multiple clinical trials [4]. However, clinical outcome was often variable with great patient‐to‐patient variation [5]. This variability may in part be attributable to varying degrees of preexisting neutralizing immunity directed at circulating hAds types [6, 7]. As a means to circumvent this, non‐human primate (nhp) Ads have been considered as an alternative source for the generation of new oncolytic Ad vectors [8]. The nhpAds of the HAdV‐B species especially have shown potent and broadly acting oncolytic potential in several tumour types including prostate, bladder, and pancreatic cancer *in vitro* [9]. In addition, no preexisting neutralizing immunity directed against these viruses could be detected in the population.

Therefore, we developed an oncolytic derivative of the gorilla‐derived HAdV‐B AdV‐lumc007 by deletion of one of the Retinoblastoma (Rb)‐binding domains in *E1A*. Rb protein is a tumour suppressor and regulates cell cycle progression to S‐phase by directly binding to the transcription factor E2F1 [10]. Deletion of this binding domain restricts Ad replication to fast‐dividing cells, resulting in tumour‐selectivity [11]. This new Ad vector named ‘GoraVir’ demonstrated superior oncolytic potential compared to human Ad type 5 (HAdV‐C5) by virtue of its faster dissemination and increased replication potential cancer cells *in vitro* [9]. However, targeting of cancer cells alone is not sufficient due to *i.e*. dense stromal tissues that make up the majority of the TME in solid tumours. Additionally, the multilayered barriers of the TME provide a complexity that is not reflected in two‐dimensional (2D) culture systems [12, 13]. As such, it remains to be explored whether GoraVir is in fact a suitable candidate for the treatment of PDAC. In this study, we characterized GoraVir's lytic potential in 2D and 3D‐culture models of PDAC and established proof‐of‐concept *in vivo*.

## 2 Materials and methods

### 2.1 Cells

Pancreatic cancer cell lines BxPC‐3 (RRID:CVCL_0186), PATU‐T (RRID:CVCL_1847), and MIA PaCa‐2 (RRID:CVCL_0428) were all purchased from the American Type Culture Collection (ATCC, Virginia, USA). Cell lines were authenticated every 2 years by the Forensic Laboratory for DNA Research, Department of Human Genetics, Leiden University Medical Centre, by short tandem repeat analyses and comparison with the STR databases. The patient‐derived human pancreatic cancer cell line FNA005 was obtained in December 2019 at the Leiden University Medical Center from a single fine‐needle biopsy [14] conform the standards set by the Declaration of Helsinki and cultured in RPMI‐1640 (Biowest, Nuaillé, France) supplemented with 8% foetal bovine serum (FBS, Invitrogen, Bleiswijk, The Netherlands) and 1% Penicillin–Streptomycin (P/S, Gibco, Bleiswijk, The Netherlands). Tumour identity was established using AmpliSeq Cancer Hotspot Gene Panel V2 genome sequencing (Thermo Fisher Scientific, Bleiswijk, The Netherlands). Use of the material was undertaken with the understanding and written consent of the subject and approved by the Medical‐Ethical Review Committee (MTCC) Leiden Den Haag Delft (LDD) under issuance protocol no. B21.073. The pancreatic stellate cell line PS‐1 was kindly provided by prof. Kocher (Queen Mary University London, London, UK) and has been described previously [15]. Primary pancreatic cancer‐associated fibroblasts (CAFs) were isolated as described previously [16] and cultured in Dulbecco's Modified Eagle's Medium (DMEM)/F‐12 (Gibco) supplemented with 8% FBS and 1% P/S. Cells were cultured in DMEM supplemented with 8% FBS and 1% P/S unless stated otherwise. All cell lines were confirmed negative for mycoplasma contamination.

### 2.2 Adenoviruses

All experiments were performed using CsCl‐purified Ad stocks. A detailed outline of the CsCl‐purification method for Ads has previously been described [9]. GoraVir is a replication‐competent vector derived from the HAdV‐B gorilla AdV‐lumc007 that carries a small deletion in one of the Rb‐binding domains of *E1A* and has been described elsewhere [9]. HAdV‐C5Δ24E3 is a replication‐competent E3‐deleted vector that carries a similar deletion in one of the Rb‐binding domains of *E1A* and has also been previously described [11].

### 2.3 Cell killing assay

For monolayer infection, cells were seeded at 1 × 10^4^ cells per well in a flat bottom 96‐well plate in normal cell culture medium and incubated o/n at an atmosphere of 5% CO_2_ at 37 °C. For the generation of monoculture spheroids, cells were seeded at 1 × 10^4^ cells per well in a 96‐well U‐bottom plate (Corning Costar, Amsterdam, The Netherlands) in culture medium supplemented with 0.25% methylcellulose (Sigma‐Aldrich, Amsterdam, The Netherlands) and centrifuged at 300 ***g*** for 1 min. Cells were cultured at an atmosphere of 5% CO_2_ at 37 °C for 2 days to allow for the formation of spheroids. On the day of infection, supernatant was removed and cells were infected at the indicated multiplicity of infection (MOI) in culture medium supplemented with 2% FBS and 1% P/S. Cell viability was measured after 5–6 days using the cell proliferation reagent kit WST‐1 (Merck, Amsterdam, The Netherlands) according to manufacturer's instructions.

### 2.4 IHC of spheroid co‐cultures

BxPC‐3 and PS‐1 cells were co‐cultured using 2.5 × 10^5^ cells per well of each cell line in a 96‐well U‐bottom plate (Corning Costar) in culture medium supplemented with 0.25% methylcellulose and centrifuged at 300 ***g*** for 1 min. Cells were incubated at an atmosphere of 5% CO_2_ at 37 °C for 2 days to allow for the formation of spheroids. Next, spheroids were collected in 15 mL tubes using a P1000 pipet. When all spheroids had drifted to the bottom of the tube, supernatant was discarded and replaced by 500 μL virus at MOI 10 in cell culture medium supplemented with 2% FBS and 1% P/S. Spheroids were plated in an ultra‐low attachment 24‐well flat bottom plate (Corning Costar) using 10–20 spheroids per well. After 5 days, spheroids were collected and paraffin‐embedded sections were used in IHC using anti‐adenovirus hexon antibody (1 : 2000) and biotinylated goat‐α‐mouse secondary antibody (1 : 200, #E0433, Agilent Technologies, CA, USA). Immunoreactivity was visualized using VECTASTAIN^®^ Elite ABC‐HRP kit (Vector Laboratories, Amsterdam, The Netherlands) and DAB substrate (DAKO, Glostrup, Denmark) according to manufacturer's instructions. Counterstain was performed using haematoxylin (Sigma‐Aldrich). Light microscopy pictures were obtained using an Olympus BX51 (Olympus Scientific Solutions, Tokyo, Japan).

### 2.5 Generation of CD46‐KO cell lines

Single guide RNAs (sgRNAs) were designed against human CD46 (forward: 5′‐CACCGAAGGAAAGGGACACTCGCGG‐3′; reverse: 5′‐aaacCCGCGAGTGTCCCTTTCCTTC‐3′) and cloned in a BsmBI‐digested plentiCRISPRv2‐puromycin vector (#98290, Addgene, Cambridge, UK) as described elsewhere [17]. Sanger sequencing was performed to verify the correct insert of the sgRNA using an U6‐promoter primer (forward: 5′‐GAGGGCCTATTTCCCATGATT‐3′). For the generation of an empty vector (EV) control cell line, a BsmBI‐digested plentiCRISPRv2‐puromycin vector was used in which no sgRNA was cloned. Subsequently, lentiviruses were generated using third‐generation packaging vectors and HEK293T cells according to standard techniques [18]. One day prior to infection 4 × 10^5^ cells of A549 or MIA PaCa‐2 were seeded per well in a 6‐well plate (Thermo Fisher Scientific) in normal cell culture medium. Next, 500 μL virus‐containing supernatant was mixed with 500 μL cell culture medium supplemented with 20 μg·mL^−1^ polybrene (Merck). Cells were transduced for 48 h and transferred to a T‐25 flask (Greiner Bio‐One, Kremsmünster, Austria) and puromycin selection (2 μg·mL^−1^, Sigma‐Aldrich) was performed. From the surviving cells which were transduced with the vector containing sgRNAs against CD46, pure CD46‐negative populations were isolated by fluorescence‐activated cell sorting (FACS) using a BD FACSAria II 3L (BD, New Jersey, USA). Isolated populations were subsequently cultured in cell culture medium supplemented with puromycin and an atmosphere of 5% CO_2_ at 37 °C. EV control cells were not sorted and cultured under similar conditions.

### 2.6 Flow cytometry

Cells were stained extracellular with primary antibodies anti‐CAR (1 : 1000, #05‐644, Millipore, Amsterdam, The Netherlands), anti‐CD46 (1 : 125, #555948, BD), or DSG‐2 PE (1 : 150, #12‐9159‐42, Invitrogen) according to standardized methods. For intracellular staining, cells were fixed using 4% paraformaldehyde at 4 °C for 20 min and permeabilized using Cytofix/Cytoperm™ (BD) before staining with anti‐adenovirus hexon (1 : 1000, #ab8251, Abcam, Cambridge, UK). As a secondary antibody goat‐α‐mouse PE (1 : 1000, #12‐4010‐82, eBioscience, Amsterdam, The Netherlands) was used. Samples were analysed using a LSR‐II cytometer (BD) and analysed using flowjo software (version 10.8.0) (BD FlowJo, Ashland, OR, USA).

### 2.7 Animal experiments

Mouse experiments were permitted by the animal welfare body (IvD) of Leiden University Medical Center and carried out under the project license AVD1160020187004 issued by the national competent authority on animal experiments (CCD), in accordance with the Dutch Act on Animal Experimentation and EU Directive 210/63/EU. Male and female nonobese diabetic (NOD).Cg‐Prkdc^scid^Il2rg^tm1Wjl^/SzJ (NSG) mice (RRID:IMSR_JAX:005557) were obtained from The Jackson Laboratory and maintained at the breeding facility of the LUMC in Leiden, The Netherlands. Mice were 12 weeks old at the start of the experiment and were housed in individually ventilated cages with no more than 4 mice per cage. For tumour challenge, low‐passage BxPC‐3 cells were harvested and 5 × 10^6^ cells in 100 μL PBS supplemented with 0.1% BSA were implanted subcutaneously. Mice body weight was monitored throughout the experiment. Tumour volume was measured three times a week using a caliper and calculated (tumour volume = width × length × height). When all tumours reached a size of 50–200 mm^3^ mice were distributed equally among the three treatment groups (*n* = 5/group) according to tumour size and gender and groups were randomly assigned to a particular treatment. Tumours were treated with PBS, GoraVir, or HAdV‐C5Δ24E3 by intratumoural injection of 1 × 10^8^ plaque‐forming units (pfu) virus in 30 μL PBS under isoflurane anaesthesia. Treatment was not blinded. Ten days after treatment, mice were sacrificed by CO_2_ inhalation and tumour, spleen, liver, and serum were collected for real‐time (RT) quantitative (q)PCR analyses.

### 2.8 Adenovirus genome copies

Genomic DNA was isolated using the Purelink™ Genomic DNA Kit (K182000, Invitrogen) according to the manufacturer's instructions and DNA concentrations were determined by NanoDrop™ 1000 Spectrophotometer (Thermo Fisher Scientific). Adenovirus genome copies were determined as described previously [9].

### 2.9 Statistical analyses

All statistical analyses were performed using graphpad prism software (v9.0.1, La Jolla, CA, USA). Data are presented as mean ± SEM unless otherwise stated. Unpaired analyses (one‐way ANOVA and unpaired *t* tests) were used for analysis of repeated experiments, and *P* < 0.05 was considered significant throughout. Detailed descriptions about statistical analysis are described in the figure legends. Significant differences are indicated by asterisks, with *P* values <0.05 shown as *, <0.01 as **, and <0.001 as ***.

## 3 Results

### 3.1 GoraVir shows strong lytic potential in cancer cells and cancer‐associated fibroblasts *in vitro*

**Fig. 1:**
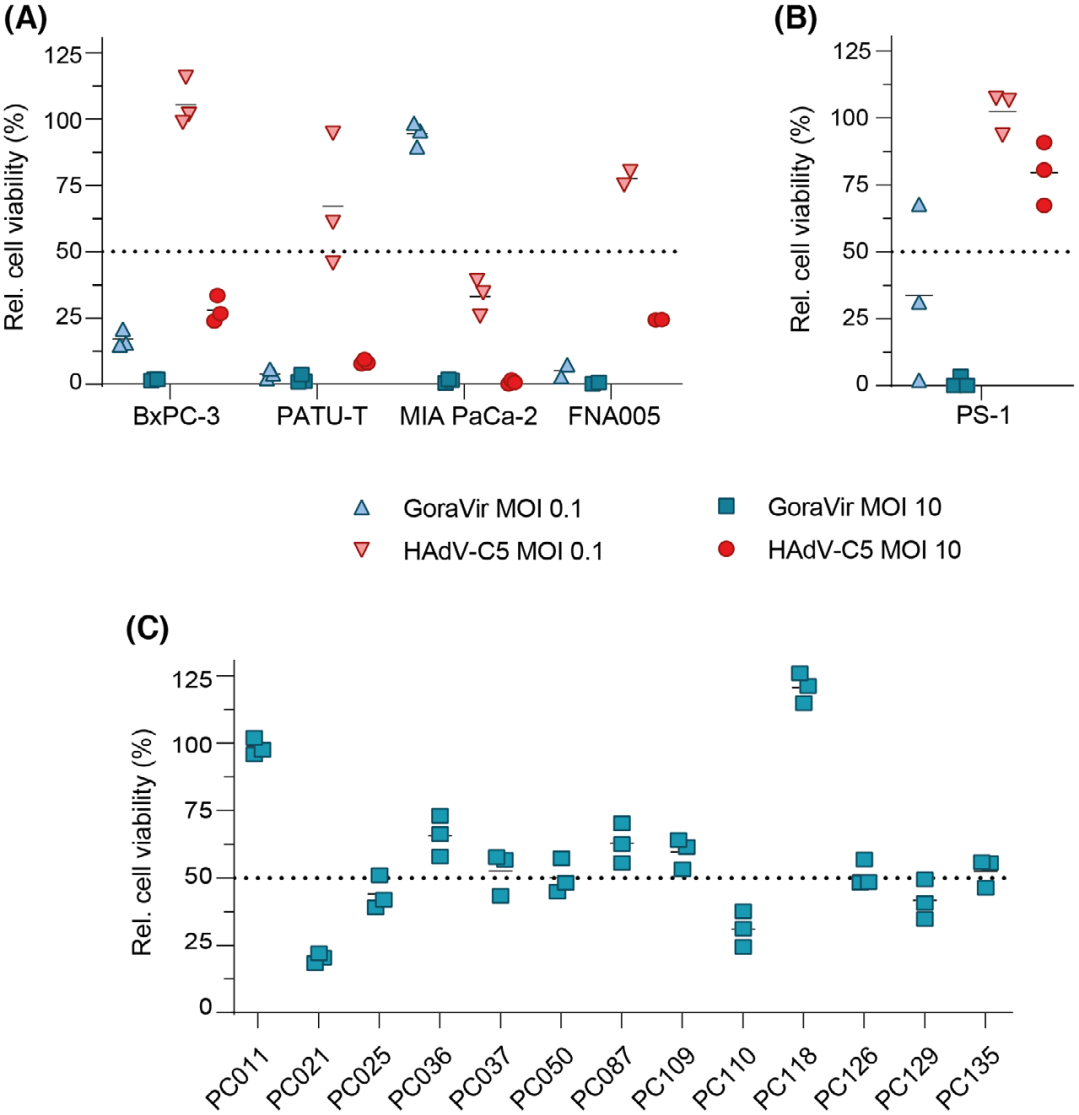
Infection of pancreatic cancer cells and cancer‐associated fibroblasts with GoraVir. (A) Pancreatic cancer cell lines BxPC‐3, PATU‐T and MIA PaCa‐2, as well as the patient‐derived pancreatic cancer cell line FNA005, and (B) the pancreatic stellate cell line PS‐1 were infected with GoraVir or HAdV‐C5 at multiplicity of infection (MOI) 0.1 or MOI 10 and cell viability was measured by WST assay at day 6 post infection. Mean is depicted relative to uninfected cells of *n* = 2 (FNA005) or *n* = 3 biological replicates each performed in triplicate; (C) Patient‐derived primary fibroblasts isolated from pancreatic cancer tissues were infected with GoraVir MOI 10 and cell viability was measured by WST assay at day 5 post infection. Mean is depicted relative to uninfected cells of a representative figure of *n* = 2 biological replicates each performed in triplicate. The dashed line denotes 50% reduction in cell viability.

GoraVir is an oncolytic derivative of the HAdV‐B AdV‐lumc007 isolated from a gorilla and demonstrated strong oncolytic potential at high MOI in an *in vitro* screening panel consisting of 29 tumour cell lines encompassing four tumour types including glioblastoma, bladder cancer, prostate cancer, and pancreatic cancer [9]. To validate GoraVir's ability to kill pancreatic cancer cells, BxPC‐3, PATU‐T, MIA PaCa‐2, and a low‐passage patient‐derived pancreatic cancer cell line (FNA005) were infected at MOI 0.1 and 10 with GoraVir or HAdV‐C5, as a reference control (Fig. 1A). Infection with GoraVir at both MOI resulted in (near‐)complete cell killing in all four cell lines at 6 days post infection, except for MIA PaCa‐2 at MOI 0.1 (mean residual viability 94.0 ± 1.52%). Infection with HAdV‐C5 also resulted in strong cell killing at MOI 10 in all cell lines although slightly less effective than GoraVir. In line with this, infection with HAdV‐C5 at low MOI resulted only in moderate cell killing. Similar observations were made when calculating the EC50 values for both viruses in these cell lines (Table S1). These data validated our previous observations that GoraVir demonstrated superior cytotoxicity in pancreatic cancer cells compared to HAdV‐C5.

In PDAC, cancer‐associated fibroblasts (CAFs) are a highly abundant cell population and linked to the establishment of an immunosuppressive TME [19]. Consequently, therapeutic strategies targeting these cells have gradually emerged [20, 21]. Recently, it was demonstrated that oncolytic mammalian orthoreovirus (reovirus) was able to infect and kill human untransformed CAFs isolated from several gastrointestinal tumour types *in vitro*, in contrast to an oncolytic derivative of HAdV‐C5 [16]. To investigate whether GoraVir does have the potential to infect and lyse CAFs, the pancreatic stellate cell line PS‐1 was exposed to GoraVir or HAdV‐C5 at high and low MOI and cell viability was determined at 6 days post infection (Fig. 1B). Similar to pancreatic cancer cells, GoraVir induced near‐complete cell killing in PS‐1 at MOI 10 and retained a strong lytic potential at a lower MOI. In contrast, HAdV‐C5 showed minor cytotoxicity at MOI 10 (mean residual viability 80.6 ± 3.58%) and no cell killing at a lower MOI. To continue our exploration of GoraVir's ability to infect and kill CAFs, we exposed patient‐derived primary fibroblasts previously isolated from pancreatic cancer tissues [16] with GoraVir at MOI 10 and measured cell viability at 5 days post infection. Again, GoraVir was shown to induce cell killing in the majority of primary fibroblasts (Fig. 1C). In fact, for 8 out of 13 fibroblast cell lines cell viability was reduced by ~ 50% or more. Taken together, it appears that GoraVir demonstrates strong lytic potential in pancreatic cancer cells as well as tumour‐associated stromal cells.

### 3.2 GoraVir productively infects and lyses 3D tumour spheroids and tumour‐fibroblast co‐cultures of PDAC

**Fig. 2:**
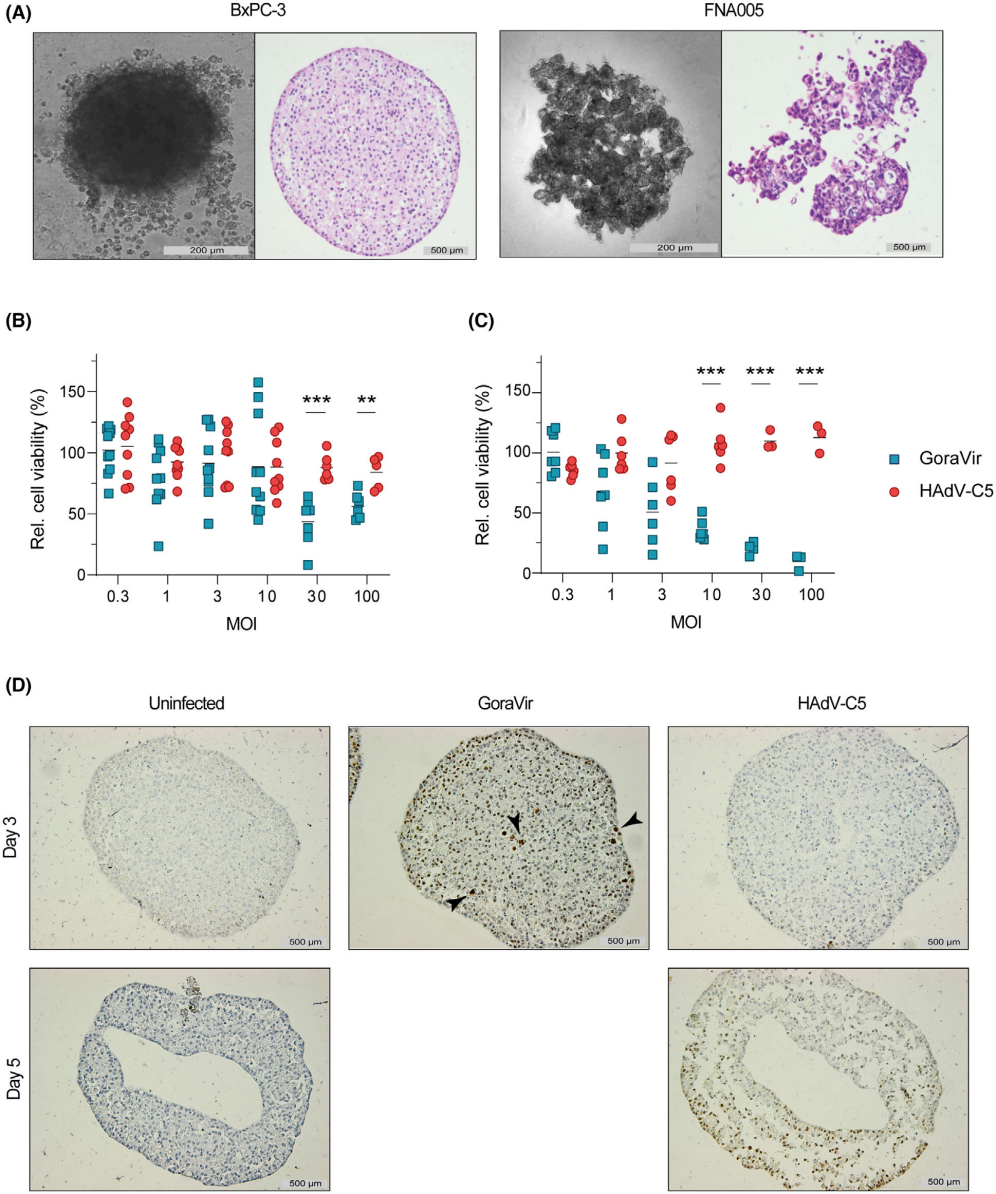
Lytic potential of GoraVir and HAdV‐C5 in PDAC spheroid cultures. (A) Light microscopy pictures of spheroid monocultures of the pancreatic cancer cell line BxPC‐3 and the patient‐derived pancreatic cancer cell line FNA005, and haematoxylin and eosin (HE) staining of co‐cultures of these cells with the stellate cell line PS‐1. Scale bar represents 200 μm (light microscopy) and 500 μm (HE) size; (B) Spheroid monocultures of BxPC‐3 or (C) FNA005 cells were infected with GoraVir or HAdV‐C5 at different multiplicity of infection (MOI) and cell viability was determined at 5 days post infection. Means are depicted relative to uninfected cells, and each symbol represents an individual spheroid. Statistical analyses were performed using multiple unpaired t tests and the Holm‐Šídák correction. Significant differences are indicated by asterisks, with *P* values < 0.01 shown as **, and <0.001 shown as ***; (D) Immunohistochemistry (IHC) staining of adenovirus hexon protein (brown) in BxPC‐3/PS‐1 co‐culture spheroids infected with virus at MOI 10 at 3 and 5 days post infection. Black arrows indicate signs of cytopathic effect (CPE). Illustrated are representative spheroids of *n* = 5. Scale bar represents 500 μm size.

Spheroid cultures of PDAC are an interesting model for *in vitro* drug testing as they resemble more closely the *in vivo* setting by mimicking important pathobiological aspects, including cell–cell and cell‐matrix interactions [13]. To validate whether GoraVir demonstrates its oncolytic potential in 3D models, we first generated monoculture spheroids of BxPC‐3 or FNA005 cells (Fig. 2A). Spheroids were infected with GoraVir or HAdV‐C5 at different MOI and cell viability was measured at 5 days post infection. In the BxPC‐3 spheroids, infection with GoraVir showed a considerable reduction in cell viability at MOI ≥ 30 (mean residual viability 43.5 ± 19.3%) although this did not increase upon higher MOI (Fig. 2B). In contrast, infection with HAdV‐C5 did not result in any cell killing at any MOI tested. Infection of the FNA005 spheroids showed a similar trend, where GoraVir‐infected spheroids demonstrated a dose‐dependent reduction in cell viability ultimately nearing complete cell killing at MOI 100 (mean residual viability 7.83 ± 6.8%) while cell viability of HAdV‐C5‐infected spheroids did not decrease (Fig. 2C). In conclusion, GoraVir demonstrated to be significantly more potent in killing monoculture tumour spheroids, similar to what was observed in monolayer cultures.

The inability of HAdV‐C5 to kill spheroid monocultures of the two cell lines could be related to a higher resistance to infection at baseline, as illustrated by the infection of monolayer cell cultures in Fig. 1. Therefore, we sought to determine whether GoraVir's superior oncolytic potential was attributable to a higher infection efficiency. Tumour‐fibroblast spheroids were generated, consisting of cancer cells and PS‐1 cells, since cancer cells alone did not allow for the formation of spheroids suitable for immunohistochemistry analyses. Similarly, the FNA005‐fibroblast spheroids were shown to disintegrate upon processing and were excluded from subsequent experiments (Fig. 2A). BxPC‐3‐fibroblast spheroids were infected with GoraVir or HAdV‐C5 at MOI 10 and stained for Ad hexon protein at 3 days post infection (Fig. 2D). Infection with GoraVir resulted in a clear penetration of the tumour‐fibroblast spheroids by the virus, illustrated by a considerable number of hexon‐positive cells. Moreover, some cells showed a cytopathic phenotype (indicated by the black arrows). Conversely, infection with HAdV‐C5 did not yield any hexon protein in the co‐culture spheroids at 3 days post infection, similar to the uninfected control. Nevertheless, hexon staining was observed at 5 days post infection with HAdV‐C5. As such, it appears that HAdV‐C5 can infect co‐culture spheroids albeit less efficiently than GoraVir. In support of this, very few GoraVir‐infected spheroids could be retrieved at day 5 post infection. The absence of intact spheroids is most likely due to the loss of integrity caused by the combined effect of the infection as well as the formation of a necrotic core after longer periods of culturing [22]. Taken together, it appears that GoraVir lytic potential in 3D culture models of PDAC can be attributed to its ability to efficiently infect and penetrate tumour spheroid cultures.

### 3.3 GoraVir uses CD46 as its primary entry receptor into human cells

The superior cytolytic efficacy of GoraVir, compared to HAdV‐C5, and its broad tropism prompted us to explore the mode of entry of this virus. For entry into the host cell, Ads bind their primary receptor via the fibre knob, followed by secondary interactions between the penton‐base proteins and integrins on the cell surface, leading to endocytosis of the virion into the host cell [23]. Ads of the HAdV‐C species make use of the coxsackie and adenovirus receptor (CAR) for entry into the host cell [24, 25]. Reduced expression of CAR has shown to be a major limiting factor in transduction efficiency and elaborate efforts have been made to retarget these vectors by e.g., replacement of the fibre knob protein [26, 27, 28, 29]. Most hAds, as well as several chimpanzee‐derived Ads, of the HAdV‐B species make use of cluster of differentiation 46 (CD46) protein as their main entry receptor into human cells [30, 31]. CD46 is a membrane protein involved in the regulation of complement deposition, ubiquitously expressed on nucleated cells, and its expression is upregulated in many tumour types [32]. Considering GoraVir's broadly acting oncolytic potential and genomic resemblance to members of the HAdV‐B species [9], we hypothesized that it uses this receptor for viral entry into the host cell. Interestingly, we observed a ~ 50 to 500‐fold higher CD46 expression mRNA expression than CAR mRNA expression levels in the patient‐derived primary fibroblasts (Fig. S1). Therefore, CD46 might pose a valuable receptor for targeting of cancer cells as well as CAFs in PDAC.

**Fig. 3:**
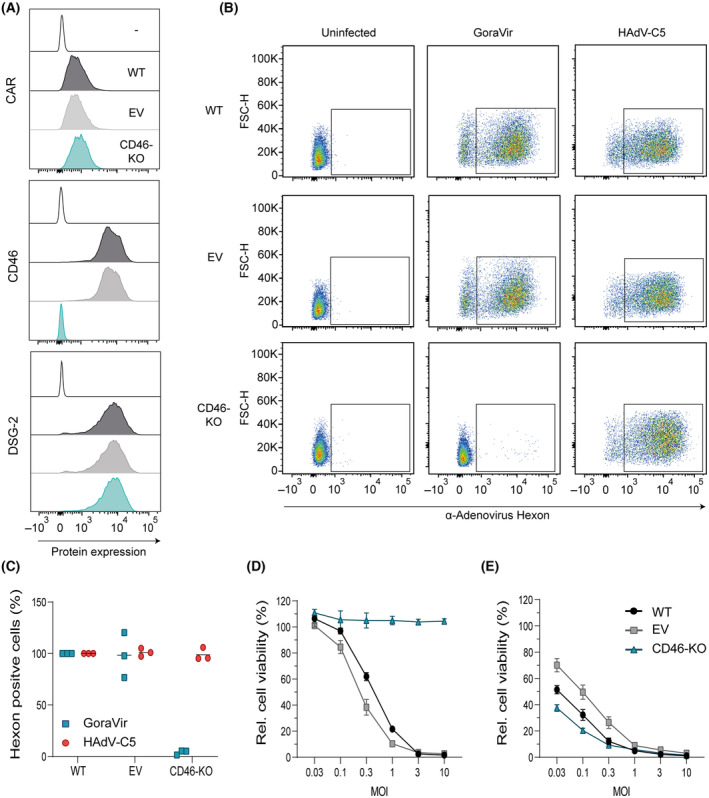
Infection of MIA PaCa‐2 CD46 knockout cells with GoraVir and HAdV‐C5. (A) Cell surface protein expression of CAR, CD46, and DSG‐2 on unstained (−), wildtype (WT), empty vector (EV) control, or CD46 knock out (KO) MIA PaCa‐2 cells; (B) WT, EV, and KO MIA PaCa‐2 cells were infected with GoraVir or HAdV‐C5 at multiplicity of infection (MOI) 10 for 48 h after which hexon protein expression was measured by flow cytometry. Depicted are representative figures of *n* = 3 biological replicates; (C) Percentage of hexon positive cells relative to WT‐infected cells. Depicted are means of *n* = 3 biological replicates; (D) WT, EV, and KO MIA PaCa‐2 cells were infected with GoraVir or (E) HAdV‐C5 at different MOI and cell viability was measured by WST assay at 6 days post infection. Depicted are mean ± SEM of *n* = 3 independent experiments each performed in triplicate.

To determine whether GoraVir indeed makes use of CD46 for entry into human cells, a stable CRISPR/Cas9‐mediated CD46‐knockout cell line was established in MIA PaCa‐2 and A549 cells. After FACS sorting the CD46‐negative population (CD46‐KO), cells were checked for the expression of the three receptors frequently used by hAds for viral entry: CAR, CD46, and desmoglein‐2 (DSG‐2) [30, 33]. Wildtype (WT) and empty vector (EV) control cells for both cell lines showed high surface expression of CD46 and DSG‐2, and to a lesser extent, CAR (Fig. 3A; Fig. S2A). While surface expression of CAR and DSG‐2 was maintained in the two CD46‐KO cell lines, no CD46 expression could be detected, thereby confirming the CRISPR/Cas9‐mediated knockout of CD46 (Fig. 3A, Fig. S2A). Next, cells were infected with GoraVir or HAdV‐C5 and hexon protein expression was measured at 24 or 48 h post infection for A549 cells (Fig. S2B) and MIA PaCa‐2 (Fig. 3B), respectively. Compared to WT and EV cells, deletion of CD46 strongly reduced the number of hexon‐positive cells upon infection with GoraVir but not HAdV‐C5, in both MiaPaCa2 and A549 cells. In fact, infection of the MIA PaCA‐2 CD46‐KO cells with GoraVir was similar to uninfected cells (Fig. 3C), suggesting the virus was no longer able to enter the cells. For A549 cells, a less prominent but significant reduction was observed in the absence of CD46 compared to WT (*P* = 0.015) and EV cells (*P* = 0.012) (Fig. S2C). One explanation why GoraVir was still able to enter A549 CD46‐KO cells might be due to the use of an alternative route of entry in these cells, as has been observed for fibre gene‐deleted HAdV‐C5 vectors [34].

To confirm that the inability to enter cells directly influenced its ability to kill these cells, all cell lines were infected with GoraVir or HAdV‐C5 at different MOI and cell viability was measured at 6 days post infection. As expected, the MIA PaCa‐2 CD46‐KO cell line showed no killing upon infection with GoraVir at any of the MOI tested (Fig. 3D). In contrast, the absence of CD46 had no inhibitory effect on cell killing with HAdV‐C5 (Fig. 3E). Similar observations were made in the A549 CD46‐KO cell line at 3 days post infection although the phenotype after infection with GoraVir was lost upon higher MOI (Fig. S2D,E). These observations are in line with the relatively high amount of virus still being able to enter the cells at MOI 10 (Fig. S2B). Nevertheless, it seems that the absence of CD46 strongly reduces GoraVir's ability to infect and kill these cancer cell lines. Moreover, the observation that infectivity was similar to uninfected cells in the MIA PaCa‐2 cell line strongly suggests that CD46 can be used as a (primary) entry receptor by GoraVir.

### 3.4 A single intratumoural dose of GoraVir delays tumour growth in a xenograft NGS mouse model

**Fig. 4:**
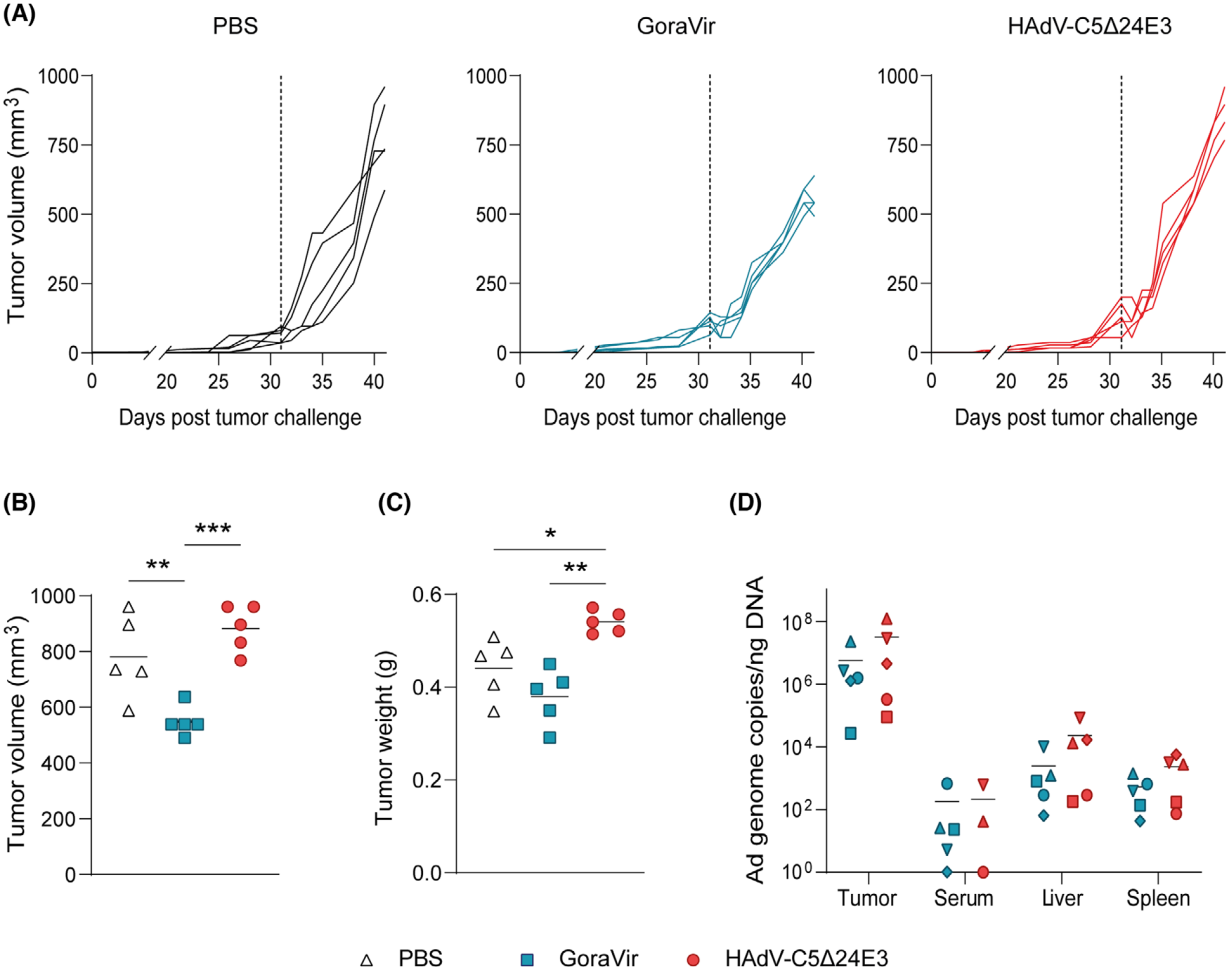
Single‐dose intratumoural injection of GoraVir or HAdV‐C5Δ24E3 in a BxPC‐3 xenograft model. (A) NSG mice were subcutaneously injected with 5 × 10^6^ BxPC‐3 cells and upon the presence of palpable tumours, PBS or 1 × 10^8^ plaque forming units of GoraVir or HAdV‐C5Δ24E3 was injected intratumourally. Depicted are the individual tumour growth curves of PBS control group, GoraVir, or HAdV‐C5Δ24E3‐treated mice (*n* = 5 per group). The dashed line denotes the start of the treatment; (B) Mean tumour volume and (C) tumour weight on day 10 post injection for PBS, GoraVir, and HAdV‐C5Δ24E3‐treated mice (*n* = 5 per group); (D) Mean Ad genome copies in tumour tissue, serum, liver, and spleen on day 10 post injection GoraVir and HAdV‐C5Δ24E3‐treated mice (*n* = 5 per group). Statistical analyses were performed using one‐way ANOVA and the Tukey correction. Significant differences are indicated by asterisks, with *P* values < 0.05 shown as *, < 0.01 shown as **, and < 0.001 shown as ***.

To continue our preclinical evaluation of GoraVir *in vivo*, we used a BxPC‐3 xenograft model and tested the virus in parallel with an oncolytic derivative of HAdV‐C5 (HAdV‐C5Δ24E3) which harbours a similar deletion in the Rb‐binding domain of *E1A* as to increase its tumour selectivity [35]. Prior to the experiment, no differences in lytic potential were observed between HAdV‐C5 and HAdV‐C5Δ24E3 in the BxPC‐3 cell line *in vitro* (data not shown). NSG mice were subcutaneously injected with BxPC‐3 cells and palpable tumours were treated intratumourally with a single dose of 1 × 10^8^ pfu virus, or PBS as a control. Tumour growth was monitored and animals were sacrificed on day 10 post treatment for further analyses. All treatments were well‐tolerated by the animals throughout the experiment (Fig. S3). In the first days after treatment with either GoraVir or HAdV‐C5Δ24E3, but not the PBS control group, a small reduction in tumour growth was observed for several mice (Fig. 4A). However, all tumours grew out exponentially thereafter. Although exponential outgrowth was observed in all groups, there seemed to be a delay in tumour growth in the GoraVir‐treated mice compared to HAdV‐C5Δ24‐treated mice or the PBS control group. Indeed, pooled analysis of the three treatments groups revealed a significant reduction in mean tumour volume in the GoraVir‐treated group (548.8 ± 53.7 mm^3^) compared to the PBS control group (781.6 ± 147.8 mm^3^, *P* = 0.010) and HAdV‐C5Δ24E3‐treated group (883.2 ± 83.4 mm^3^, *P* < 0.001) at day 10 post treatment (Fig. 4B). Interestingly, tumour volume of HAdV‐C5Δ24‐treated tumours was somewhat increased compared to PBS‐treated tumours although this was not statistically significant. Measurements of tumour weight followed a similar trend with a reduction in mean tumour weight in the GoraVir‐treated group (0.380 ± 0.061 g) compared to the PBS control group (0.441 ± 0.064 mm^3^, *P* = 0.200) and HAdV‐C5Δ24E3‐treated group (0.541 ± 0.024 mm^3^, *P* = 0.001). Interestingly, tumour weight of HAdV‐C5Δ24E3‐treated tumours was significantly increased compared to PBS‐treated tumours (*P* = 0.028).

To determine whether the observed reduction in tumour volume and tumour weight in GoraVir‐treated mice compared to HAdV‐C5Δ24E3‐treated mice was associated with a difference in viral replication, the number of viral genomes was determined in the tumour tissues. PBS‐treated tissues were used as a baseline. Interestingly, no differences in viral genome copies were found in tumours of GoraVir and HAdV‐C5Δ24E3‐treated mice (Fig. 3D). Likewise, similar levels were observed in other sites inherent to Ad biodistribution in mice including serum, liver, and spleen, collected at the time of sacrifice [29, 36]. Note, however, that in 1/3 serum samples of HAdV‐C5Δ24E3‐treated mice no Ad DNA could be detected (two samples did not yield enough DNA for RT‐qPCR analysis) as well as 1/5 samples of GoraVir‐treated mice. Taken together, it seems that the observed delay in tumour growth upon administration of a single intratumoural dose of GoraVir might not be attributable to increased viral replication compared to HAdV‐C5Δ24E3. Therefore, it remains to be established which viral (or cellular) mechanisms underlie the observed differences between the two viruses and enable GoraVir to maintain lytic potential *in vivo*.

## 4 Discussion

Oncolytic viruses demonstrate to constitute a new treatment approach which has the potential to transform the inaccessible, immunosuppressive TME that is characteristic of PDAC. Here, we studied the oncolytic potential of GoraVir, a new gorilla‐derived oncolytic Ad, in various *in vitro* model systems of PDAC and established proof‐of‐concept of oncolysis *in vivo*. In 2D cell cultures, GoraVir showed strong lytic potential in both cancer cells as well as CAFs (Fig. 1). Moreover, GoraVir retained its lytic potential in 3D spheroid culture systems of PDAC in contrast to HAdV‐C5 (Fig. 2). Dual targeting of tumour as well as stromal compartments improves virus replication and spread and has shown to promote antitumour activity [37, 38]. However, few oncolytic viruses demonstrate natural stroma targeting. This has resulted in the generation of several genetically modified or bioselected variants with enhanced targeting to these compartments [38]. To our knowledge, this is the first report of an oncolytic adenovirus that naturally targets human primary pancreatic CAFs. Previously, the oncolytic adenovirus ICOVIR15 was shown to infect and kill fibroblast activation protein (FAP)^+^ fibroblasts in a murine glioblastoma model [39]. Another approach to target CAFs using ICOVIR15 included the incorporation of a bispecific T‐cell engager directed at FAP [40]. Meanwhile, a recent paper by Harryvan et al. [16] showed that the more wildtype‐like HAdV‐C5Δ24 was unable to induce cell death in human primary CAFs in contrast to wildtype and bioselected mutant reoviruses. Since the resistance to Ad infection was also observed in CAR‐expressing fibroblasts, the authors attributed these differences to the Δ24‐modification in the HAdV‐C5Δ24 vector, which makes it not suitable for infecting and killing of quiescent cells with an intact Rb‐pathway. However, GoraVir harbours a similar deletion in *E1A* and demonstrated considerable potential to kill primary fibroblasts (Fig. 1B). This could indicate that CAFs do support productive infection of Rb‐dependent oncolytic Ads. Furthermore, the observation that patient‐derived primary fibroblasts express higher levels of CD46 mRNA than CAR mRNA led us to speculate the use of a differential receptor, or the expression levels thereof, influences the ability of these viruses to kill CAFs. Future studies examining the ability of the various Ad types to infect CAFs might provide insight in the mechanisms that underly the differences in lytic potential of these Ads in the CAFs.

As anticipated, GoraVir was shown to make use of CD46 for entry into the host cell (Fig. 3). CD46 is a membrane protein involved in regulation of the complement and is frequently upregulated in cancer cells to primarily evade antibody‐mediated cytotoxicity [41]. The ubiquitous expression of this receptor and its increased expression in many tumours would make it a preferred natural entry receptor of oncolytic Ads [32]. Interestingly, retargeting of HAdV‐C5 to CD46 has previously demonstrated to enhance anti‐tumour efficacy compared to wildtype virus despite comparable protein expression of CAR and CD46 in the tumour [42, 43]. Taken from this, it appears that targeting of CD46 brings additional benefits despite its ubiquitous expression on cells. CD46 has not been demonstrated to play a direct role in cell death although intracellular complement activation can regulate cell survival via metabolic processes (reviewed in refs [41, 44]). Alternatively, in bladder cancer cells, retargeting of adenovirus to CD46 was associated with increased transduction efficacy and subsequent cytotoxicity [42]. Hence, the use of CD46 might simply result in faster viral entry which provides these viruses with a head start. Nevertheless, it remains to be established whether the differences between GoraVir and HAdV‐C5 are indeed (partially) receptor‐dependent.

Finally, we addressed whether the superior cytotoxicity of GoraVir *in vitro* also translated to increased oncolytic activity *in vivo*. In these experiments, we opted for intratumoural injection instead of intravenous delivery as this provides a fair comparison of GoraVir and HAdV‐C5Δ24E3 given the markedly distinct expression patterns of CAR and CD46 in mice. A single‐dose intratumoural injection of GoraVir indeed delayed tumour growth compared to PBS‐treated and HAdV‐C5Δ24E3‐treated mice (Fig. 4). The significant reduction in tumour volume at 10 days post treatment seems promising, as anti‐tumour effects of oncolytic viruses in pancreatic xenograft models have frequently been observed at later time points [45, 46]. Moreover, a similar dose of HAdV‐C5Δ24E3 did not show any anti‐tumour activity in our model. Interestingly, no differences in viral genome copies were found in tumour, serum, liver, and spleen between the two viruses upon sacrifice. A possible explanation for the absent correlation between viral replication and anti‐tumour activity could be that murine CAR can function as a receptor for hAds, unlike murine CD46, thereby increasing the relative amount of (non‐cancerous) cells that might support limited replication of HAdV‐C5 [47]. Alternatively, deletion of the entire E3 gene region in HAdV‐C5Δ24E3 might have crippled the virus' lytic potential due to the loss of the adenovirus death protein (ADP) [48]. It should be noted that the use of an immunodeficient mouse model to assess anti‐tumour efficacy in this study does not accurately address the interplay of the oncolytic virus with the host immune system. Importantly, this interplay is considered a major determining factor for the anti‐tumour efficacy of oncolytic viruses. In support of this, oncolytic virus monotherapy frequently generates modest results in patients while combination therapies using other immunotherapies were demonstrated to be much more effective [49]. Consequently, the anti‐tumour effects of GoraVir demonstrated here are likely an underestimation of its true potential. However, strong lytic potential does not always confer the induction of adequate anti‐tumour immune responses and may greatly vary between different OVs [50]. Therefore, it would be of interest to determine whether GoraVir induces cancer cell death that will mediate such anti‐tumour immune responses.

## 5 Conclusion

In conclusion, we have shown that GoraVir has strong lytic potential in pancreatic cancer cells and pancreatic CAFs and that this is facilitated by its use of CD46 for viral entry. Moreover, GoraVir demonstrated superior oncolytic efficacy in spheroid culture models of pancreatic cancer and displayed enhanced anti‐tumour activity *in vivo* compared to HAdV‐C5. We propose that the enhanced anti‐tumour effects are at least in part attributable to the use of different entry receptors by these viruses, although additional aspects of virus biology should not be disregarded. Regardless, our work demonstrates that GoraVir exhibits unique oncolytic properties and seems a promising candidate for the treatment of PDAC through targeting of tumour cells and tumour‐adjacent stroma.

## Conflict of interest

STFB, VK, and RCH are named inventors of patents pertaining to the use of human‐ and non‐human primate‐derived adenoviruses as viral vectors or as oncolytic agents. RCH received and receives research funds from Janssen Vaccines & Prevention B.V. (Leiden, Netherlands) for projects on adenoviruses. The funders had no role in the design of the study; in the collection, analyses, or interpretation of data; in the writing of the article; or in the decision to publish the results. The authors declare no other conflict of interests and have no other relevant affiliation or financial involvement with any organization or entity with a financial interest in or financial conflict with the subject matter or materials discussed in the article, apart from those disclosed.

## Author contributions

STFB conceived the outline of the project. STFB, TJH, and CG designed the experiments. STFB, TJH, CG, PK, and VK conducted the experiments. STFB acquired and analysed the data. STFB, TJH, and RCH wrote the article. NvM and RCH interpreted the data and critically revised the article. All authors read and approved the final article.

## Supporting information


**Fig. S1.** Infection of A549 CD46 knockout cells with GoraVir and HAdV‐C5.

**Fig. S2.** mRNA expression of CAR and CD46 in patient‐derived primary fibroblasts.

**Fig. S3.** Mice body weight upon treatment with PBS, GoraVir, or HAdV‐C5Δ24E3.

**Table S1.** EC50 values for GoraVir and HAdV‐C5 in pancreatic cancer cells and cancer‐associated fibroblasts.

## Data Availability

The data that support the findings of this study are available from the corresponding author upon reasonable request.
