# Supplementary figures and images for: Preclinical evaluation of the gorilla‐derived HAdV‐B AdV‐lumc007 oncolytic adenovirus ‘GoraVir’ for the treatment of pancreatic ductal adenocarcinoma
Source: Mol Oncol. 2024 Jan 24;18(5):1245–58. doi: 10.1002/1878-0261.13561 (PMC11076997; doi:10.1002/1878-0261.13561)

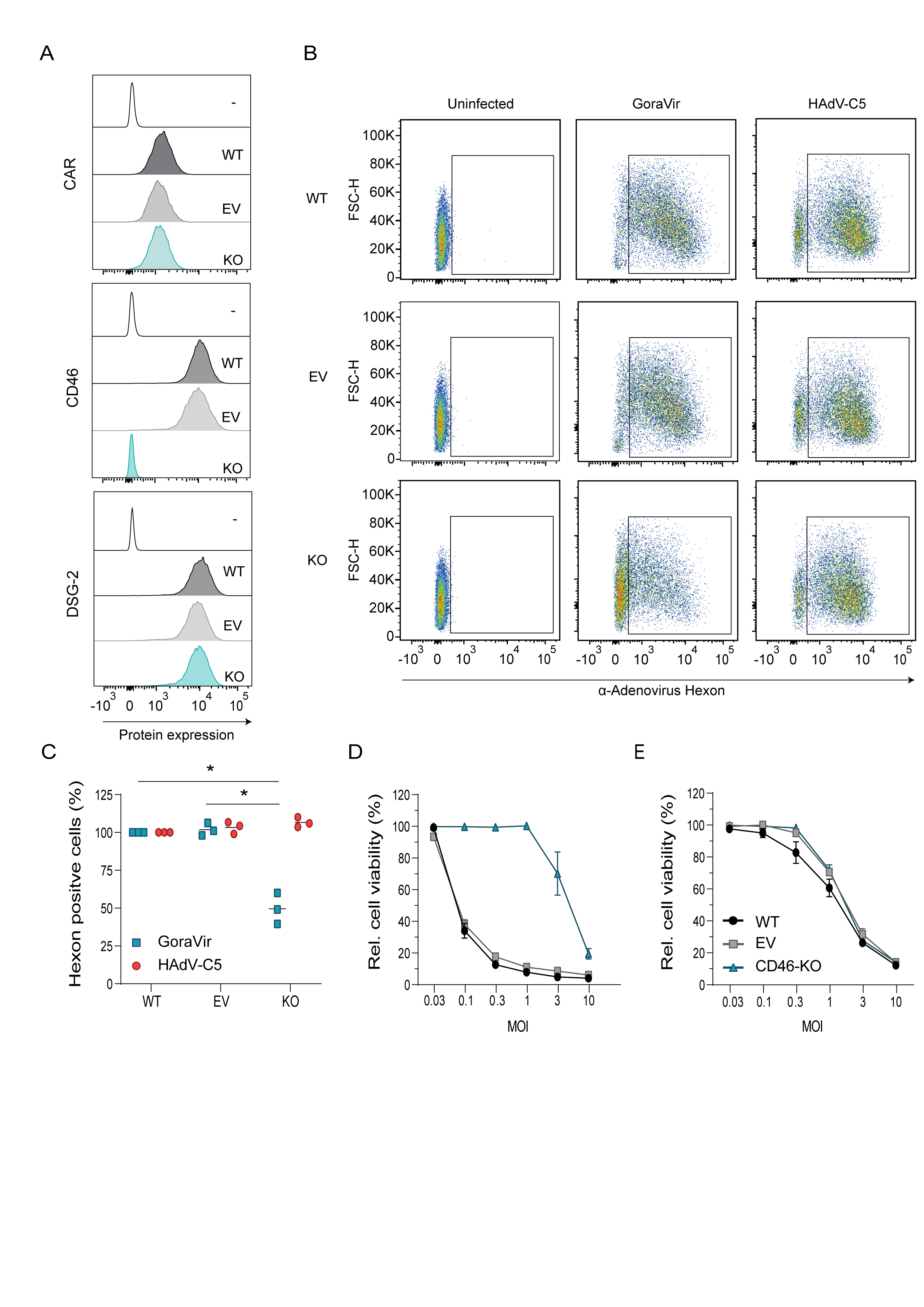

Supplement: Supplementary file 1 — Fig. S1. Infection of A549 CD46 knockout cells with GoraVir and HAdV‐C5. [file MOL2-18-1245-s003.png]

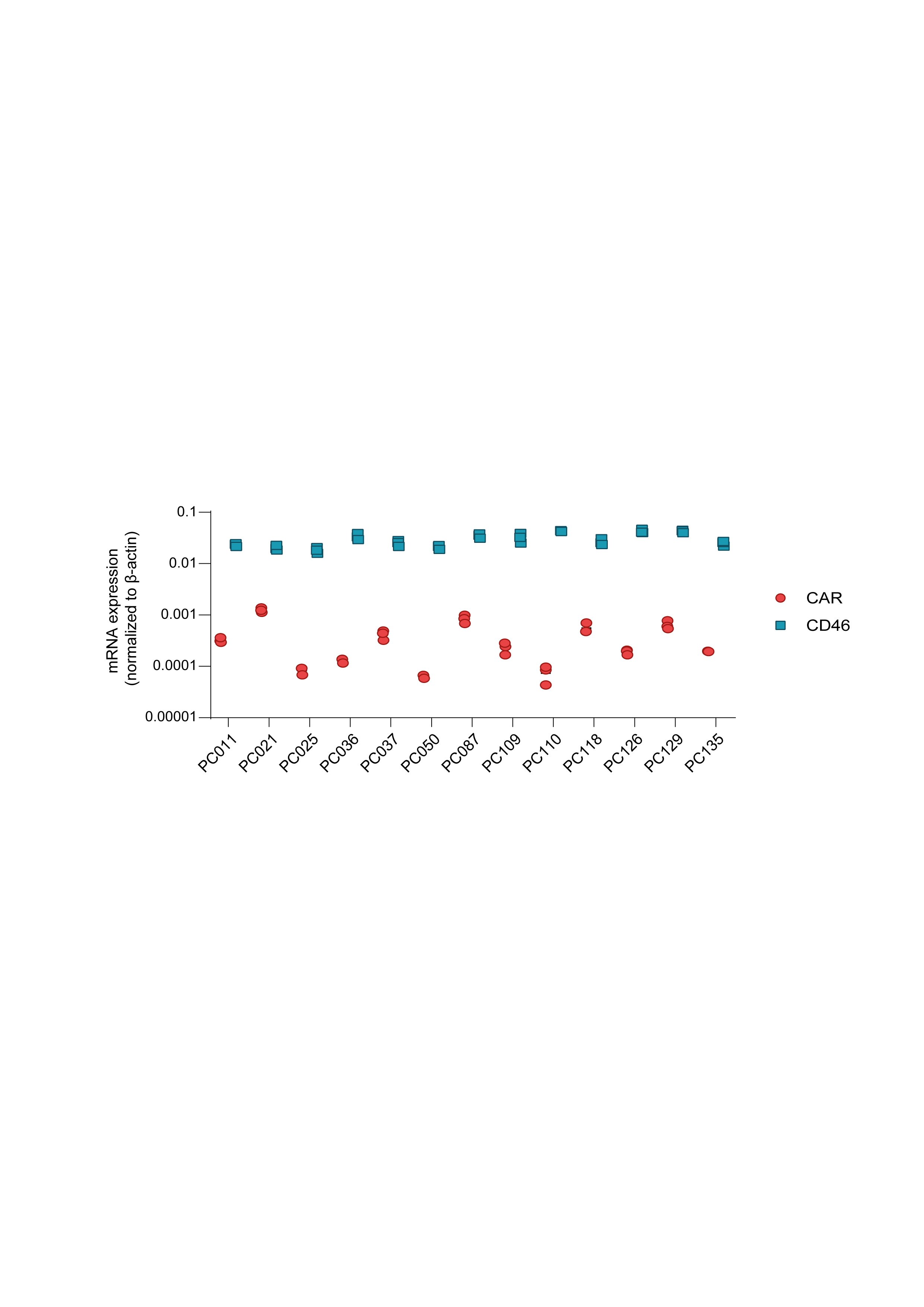

Supplement: Supplementary file 2 — Fig. S2. mRNA expression of CAR and CD46 in patient‐derived primary fibroblasts. [file MOL2-18-1245-s004.png]

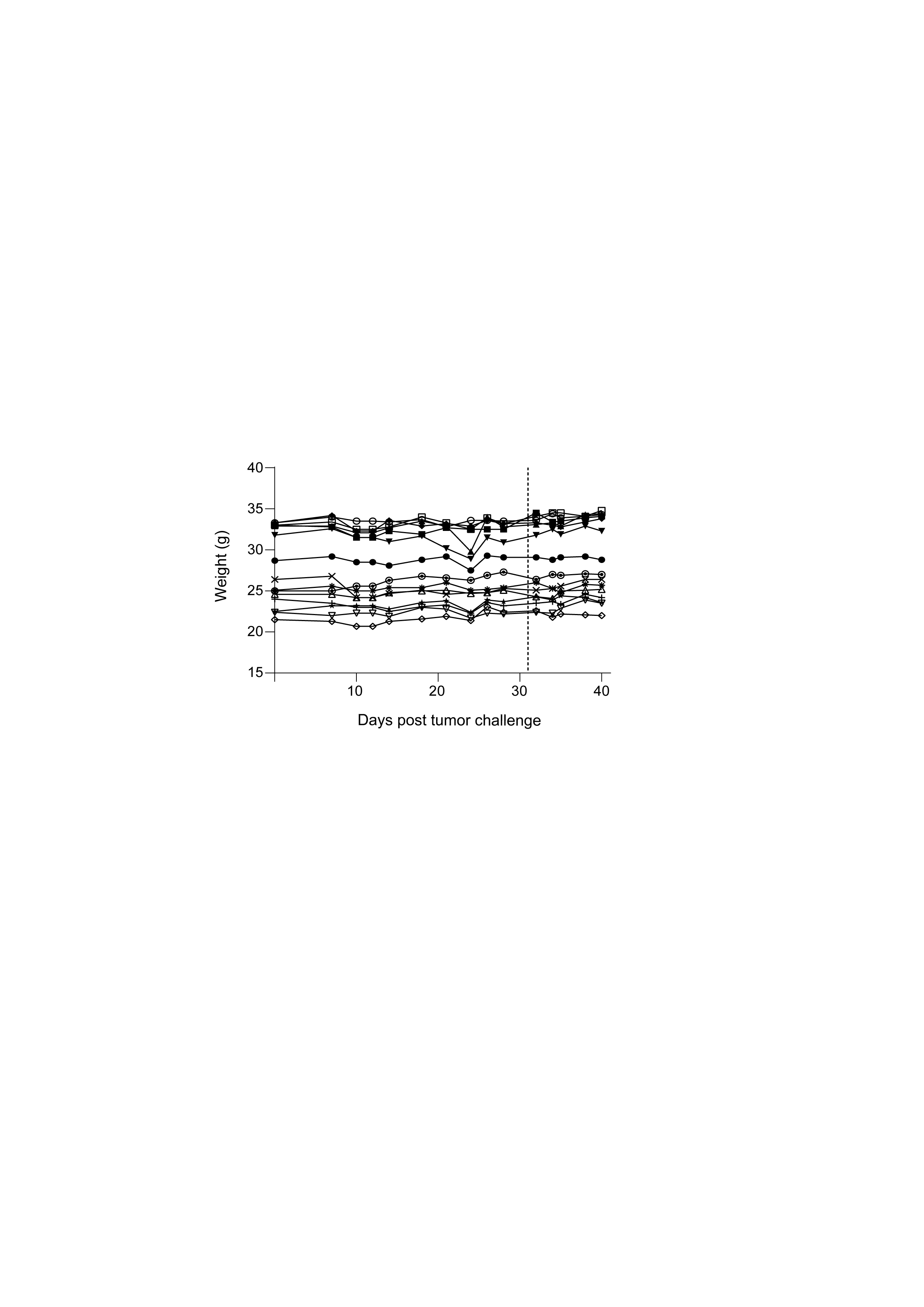

Supplement: Supplementary file 3 — Fig. S3. Mice body weight upon treatment with PBS, GoraVir, or HAdV‐C5Δ24E3. [file MOL2-18-1245-s001.png]
