# Supplementary material for: Preclinical evaluation of the gorilla‐derived HAdV‐B AdV‐lumc007 oncolytic adenovirus ‘GoraVir’ for the treatment of pancreatic ductal adenocarcinoma
Source: Mol Oncol. 2024 Jan 24;18(5):1245–58. doi: 10.1002/1878-0261.13561 (PMC11076997; doi:10.1002/1878-0261.13561)
Supplement: Supplementary file 4 — Table S1. EC50 values for GoraVir and HAdV‐C5 in pancreatic cancer cells and cancer‐associated fibroblasts. [file MOL2-18-1245-s002.pdf]

Supplementary Table S1. EC_{50} values for GoraVir and HAdV-C5 in pancreatic cancer cells and cancer-associated fibroblasts.

**Cell line**	**EC_{50}**	
	**GoraVir**	**HAdV-C5**
BxPC-3	0.021	5.403
PATU-T	<0.03[1]	0.255
MIA PaCa-2	0.444	0.036
FNA005	<0.03[1]	0.891
PS-1	0.098	19.057

[1]Complete cell-killing was still observed at the lowest concentration tested (MOI 0.03)
